# Voluntary Insurance by Patients

**Published:** 1920-07-24

**Authors:** 


### The Hospital

The Workers' Newspaper of Administrative Medicine and Institutional Life, Administration, National Insurance and Health.

No. 1781, Vol. LXVIII. SATURDAY, JULY 24, 1920. PRICE TWOPENCE

### VOLUNTARY INSURANCE BY PATIENTS.

One of the foremost points of Sir Arthur Stanley's scheme for improving the finances of the voluntary hospitals, following upon the figures revealed in Sir Napier Burnett's report on the Hospital Survey, is a more widespread endeavour to bring home to the patients the justice of a system under which they help towards the cost of their maintenance, medicines, etc. It is very desirable that some uniform procedure should be undertaken in this method of collecting patients' payments, and we have already given much publicity to the subject of organisation of employees' subscriptions, although the subscription under present conditions is not the same thing as a contribution towards the cost of treatment at the time that treatment is given.

When any carefully prepared system is available for consideration it should be examined in the light of the possibility that at least some of its salient features, if not the scheme in its entirety, may be selected with a view to incorporation in the measure universally adopted. We, therefore, have much pleasure in giving publicity (see page 431) to the outline of a scheme under which patients' contributions largely provide for the maintenance of hospitals on the voluntary principle, which has been devised by Mr. G. G. Panter, the Secretary of the Great Northern Central Hospital, whose energy and that of his Committee during recent years have been a noted feature of hospital activities in the Metropolis. The scheme is really one of insurance, and persons who come within the present scope of the voluntary hospitals may under this system secure for themselves by periodical payments hospital treatment as in or out patients. A chief feature is that the patients shall have the choice of the institution at which they may obtain treatment. We understand that the figures detailed are based upon known hospital statistics and that the recent report of the Commission on Industrial Insurance has been the guide as to the number of persons who would come within the system.

Naturally, many difficulties will arise, and, if under the new Health Services Act the Minister of Health has extended the range of State Insurance to dependants, it may be possible that the fact would conflict with the systematic collection of premiums through insurance companies, which is a point of Mr. Panter's system upon which we take it success or failure of the whole scheme will depend.

Another matter which readily occurs to one is the impossibility under present conditions of fixing the cost of an in-patient or an out-patient. Where we see, according to the annual reports of hospitals published this year, that the cost per occupied bed in many instances is somewhere between £4 and £5 per week, the valuation of £160 per annum appears to be on the low side. This, however, is a phase of economics which no one at present can accurately judge. The cost may possibly go up, although the indications are that with widespread unemployment, wages, which are the main factor in prices of all kinds, are likely to fall, and in falling bring down with them the general cost of living.

Another point is the position of the medical man. It is quite possible that the consultant would have no objection to the scheme as devised, but the general practitioner, unless he has free access to his patient from first to last, as is proposed by the Advisory Committee under the Ministry of Health, will not be in love with a system under which his patients are far more than at present taken away to voluntary hospitals.

These, however, are matters of detail, and Mr. Panter's clever scheme deserves much closer scrutiny than we have yet been able to give to it. We hope that it will give rise to an interchange of views, not only in its own regard, but as to the general principle of a universal plan of collecting contributions from patients. Naturally, in such a wide-reaching organisation as this plan of voluntary insurance, many points must arise which conflict in a smaller or greater measure with vested interests and old traditions, although the author considers that the hospital system as it is at present will be unchanged, except as regards natural developments.

